# Supplementary material for: Yttrium Doping Effects on Ferroelectricity and Electric Properties of As-Deposited Hf1−xZrxO2 Thin Films via Atomic Layer Deposition
Source: Nanomaterials (Basel). 2023 Jul 27;13(15):2187. doi: 10.3390/nano13152187 (PMC10421259; doi:10.3390/nano13152187)
Supplement: Supplementary file 1 [file nanomaterials-13-02187-s001.zip › nanomaterials-2494935-supplementary.pdf]

Supplementary Materials

Yttrium Doping Effects on Ferroelectricity and Electric Properties of As-deposited $Hf_{1-x}Zr_xO_2$ Thin Films via Atomic Layer Deposition

Youkyoung Oh[1], Seung Won Lee[1], Jeong-Hun Choi[1], Seung-Eon Ahn[2], Hyo-Bae Kim[1,]* and Ji-Hoon Ahn[1,]*

[1] Department of Materials Science and Chemical Engineering, Hanyang University, Ansan, Gyeonggi-do, 15588 Republic of Korea
[2] Department of Nano & Semiconductor Engineering, Tech University of Korea, Siheung, Gyeonggi-do, 15073 Republic of Korea
* Correspondence: hbkim9510@hanyang.ac.kr (H.-B.K.); ajh1820@hanyang.ac.kr (J.-H.A.)

1. XPS studies

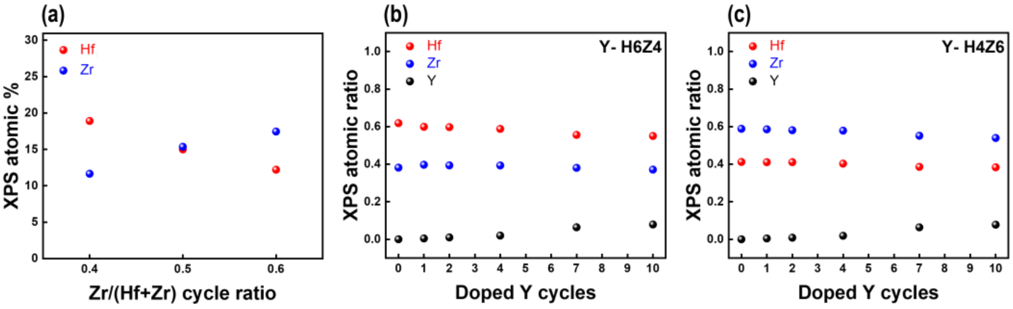

Figure S1. (a) X-ray photoelectron spectroscopy (XPS) atomic percent of Hf and Zr. XPS atomic ratio of Hf, Zr and Y in (b) as-deposited Y-H6Z4 thin films (c) as-deposited Y-H4Z6 thin films according to Y-doping cycle.

2. XRD studies

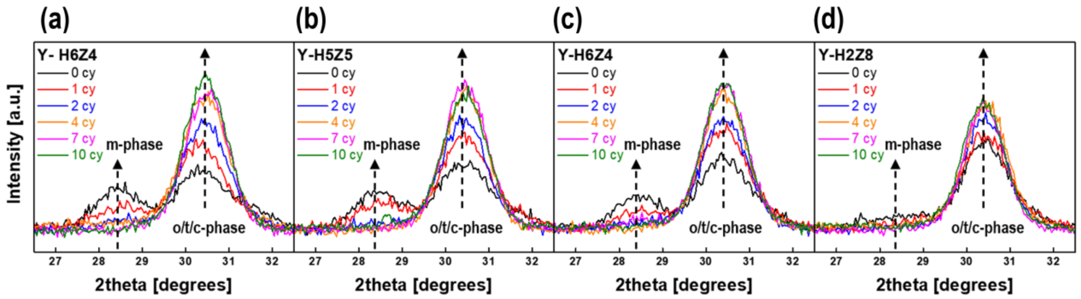

Figure S2. Grazing-angle incidence X-ray diffraction (GI-XRD) spectra of (a) as-deposited Y-H6Z4 thin films, (b) as-deposited Y-H5Z5 thin films, (c) as-deposited Y-H4Z6 thin films and (d) as-deposited Y-H2Z8 thin films according to Y-doping cycle.

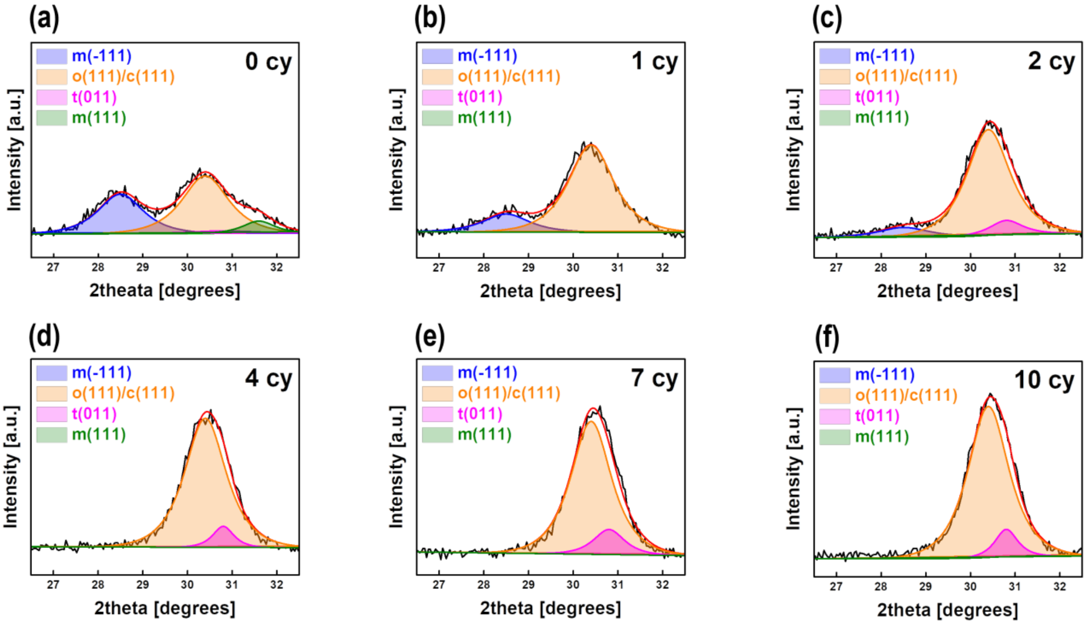

Figure S3. Peak deconvolution of grazing-angle incidence X-ray diffraction (GI-XRD) spectra of as-deposited Y-H6Z4 thin films with Y-doping cycle of (a) 0, (b) 1, (c) 2, (d) 4, (e) 7, (f) 10.
